# Determination of the optimal method for the field-in-field technique in breast tangential radiotherapy

**DOI:** 10.1093/jrr/rrt233

**Published:** 2014-02-16

**Authors:** Hidekazu Tanaka, Shinya Hayashi, Hiroaki Hoshi

**Affiliations:** Department of Radiology, Gifu University Hospital, Yanagido 1-1, Gifu 501-1194, Japan

**Keywords:** breast cancer, breast radiotherapy, breast-conserving surgery, field-in-field technique, tangential radiotherapy

## Abstract

Several studies have reported the usefulness of the field-in-field (FIF) technique in breast radiotherapy. However, the methods for the FIF technique used in these studies vary. These methods were classified into three categories. We simulated a radiotherapy plan with each method and analyzed the outcomes. In the first method, a pair of subfields was added to each main field: the single pair of subfields method (SSM). In the second method, three pairs of subfields were added to each main field: the multiple pairs of subfields method (MSM). In the third method, subfields were alternately added: the alternate subfields method (ASM). A total of 51 patients were enrolled in this study. The maximum dose to the planning target volume (PTV) (Dmax) and the volumes of the PTV receiving 100% of the prescription dose (V100%) were calculated. The thickness of the breast between the chest wall and skin surface was measured, and patients were divided into two groups according to the median. In the overall series, the average V100% with ASM (60.3%) was significantly higher than with SSM (52.6%) and MSM (48.7%). In the thin breast group as well, the average V100% with ASM (57.3%) and SSM (54.2%) was significantly higher than that with MSM (43.3%). In the thick breast group, the average V100% with ASM (63.4%) was significantly higher than that with SSM (51.0%) and MSM (54.4%). ASM resulted in better dose distribution, regardless of the breast size. Moreover, planning for ASM required a relatively short time. ASM was considered the most preferred method.

## INTRODUCTION

Most patients with early-stage breast cancer are given breast-conserving treatment consisting of wide excision and postoperative radiotherapy. Postoperative radiotherapy reduces the risk of local recurrence and results in long-term survival similar to that obtained with mastectomy [1–3]. Thus, postoperative breast tangential radiotherapy is a standard treatment. In recent years, the field-in-field (FIF) technique has become a widely preferred method for administering tangential whole breast radiotherapy. Several studies have reported that use of the FIF technique facilitates better control of dose homogeneity. The FIF technique was reported to be useful in reducing hot regions as well as cold regions [4–15]. However, the methods used for the FIF technique in these studies were not identical. There were no reports of comparisons among FIF techniques. The optimal methods to be used with the FIF technique have not been established. In this study, we classified the methods used for the FIF technique into three categories. We then simulated a radiotherapy plan using each method and analyzed the outcomes.

## MATERIALS AND METHODS

**Fig. 1.**
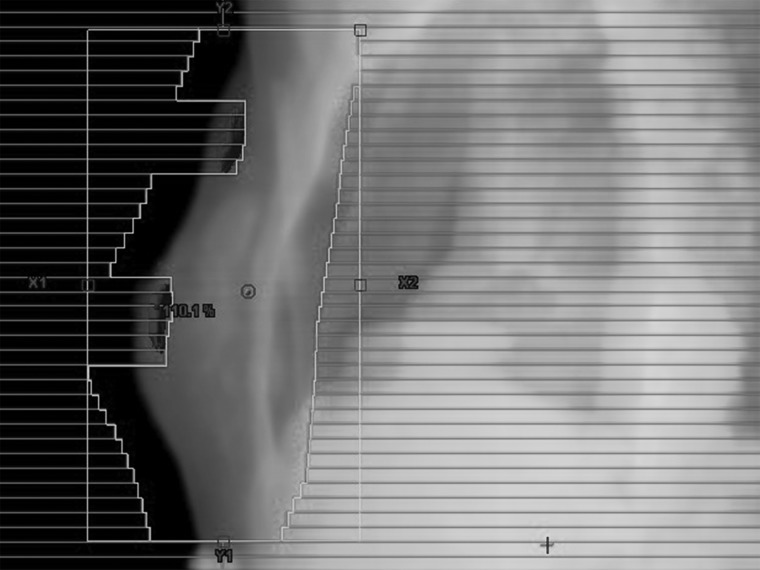
Beam's eye view for typical subfield. The subfield was manipulated to shield the areas of the breast receiving any dose cloud.

Between April 2011 and March 2013, 51 patients with early-stage breast cancer were enrolled in this planning study. All patients provided informed written consent. All patients had undergone breast-conserving surgery. Computed tomography (CT) images were obtained using a scanner with 16 detector arrays (LightSpeed Xtra; GE Healthcare, Waukesha, WI, USA) while patients were in the supine position on a breast board with both arms above their heads. Scanning was performed in 2.5-mm slices from the clavicle to the mid-abdomen during free breathing. All CT images were transferred to Eclipse External Beam Planning 8.6 (Varian Medical Systems Palo Alto, CA, USA). The ipsilateral whole breast was contoured as the clinical target volume (CTV). The planning target volume (PTV) was constructed by adding 5-mm margins and editing 5-mm of the build-up region from the skin surface of the breast. Two opposed tangential fields were set up without wedges, and the gantry angles were optimized. Leaf margins of 2 cm were added to the skin side and leaf margins of 3 mm to the other sides. Each patient's plan was normalized to a reference point at the interface of the breast and pectoralis major muscle at the level of the nipple. The prescribed dose was 50 Gy in 25 fractions. The dose calculation algorithm used was based on the pencil-beam convolution method, and the Batho power-law method was used to correct for tissue inhomogeneity. The energy of the beam was 6 MV. For FIF planning, the main field was copied as the subfield and the multileaf collimators (MLCs) were manipulated to shield the areas of the breast receiving any dose (Fig. 1). However, MLCs were not allowed to block within 1 cm of the reference point. The beam weight of the subfield was ∼ 1/10–1/15 of the main field. The minimum monitor unit (MU) of each subfield was 5. The methods used for the FIF technique were classified into three categories. In the first method, a pair of subfields was added to each main field: the single pair of subfields method (SSM). In the second method, three pairs of subfields were added to each main field: the multiple pairs of subfields method (MSM). In the third method, subfields were alternately added: the alternate subfield method (ASM).

### The single pair of subfields method

In the SSM, each main field was copied as a pair of subfields. The MLCs were manipulated to shield the areas of the breast receiving any dose (mainly at 105–107% of the prescription dose). The dose to shield the MLCs was determined such that the isodose cloud was separated from the reference point by more than 1 cm on the beam's eye view (BEV). This method was composed of four fields, including the main fields.

### The multiple pairs of subfields method

By using the same technique as that for the SSM, three pairs of subfields were generated. The MLCs were set to block the dose level at 1–2% lower than the maximum dose (Dmax), and this was followed by a 3–5% dose reduction. This method comprised eight fields, including the main fields.

### The alternate subfields method

First, the medial main field was copied as the first subfield. The MLCs were set to block the dose level at 1–2% lower than the Dmax. Then, dose calculation was performed. The beam weight of this subfield was added until the dose cloud disappeared. Second, the lateral main field was copied as the second subfield. The MLCs were set to block the dose level at 2–3% lower than the dose blocked at the first subfield. Dose calculation was performed again, and the beam weight of this subfield was added until the dose cloud disappeared. Finally, the medial main field was copied again as the third subfield. The MLCs were set to block the dose level at 2–3% lower than the dose blocked at the second subfield. After recalculation, the beam weight of this subfield was added until the dose cloud disappeared. This method was comprised of five fields, including the main fields.

The Dmax to the PTV and the volumes of the PTV receiving 100% and 95% of the prescription dose (V100% and V95%, respectively) were calculated. The homogeneity index (HI) was calculated as follows: HI = (D2 − D98)/Dprescription, where D2 is the dose administered to 2% of the PTV, D98 is the dose administered to 98% of the PTV, and Dprescription is the prescription dose. These parameters were analyzed with multiple comparison tests using the Steel–Dwass method.

**Fig. 2.**
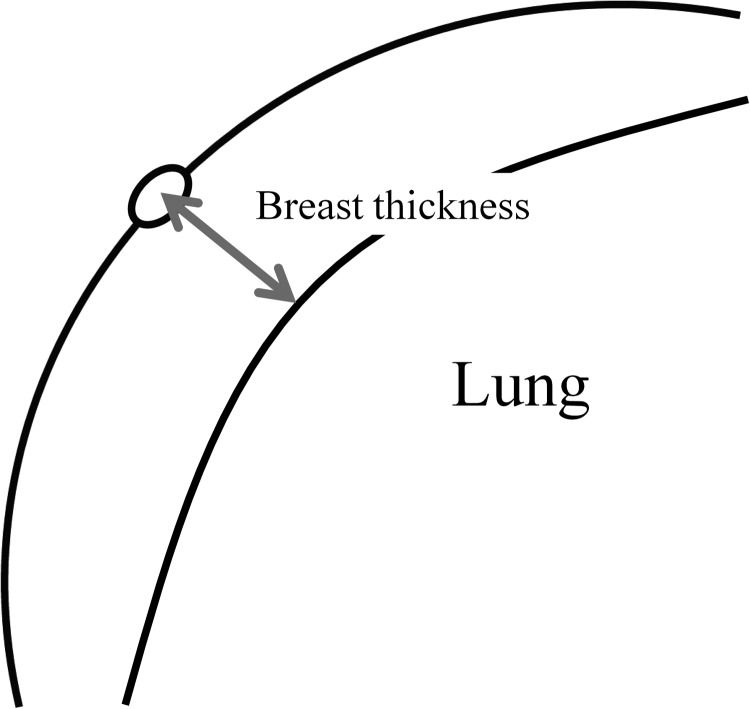
The thickness of the breast between the chest wall and the skin surface at the level of the nipple was measured.

The thickness of the breast between chest wall and skin surface at the level of the nipple was measured (Fig. 2), and patients were divided into two groups according to the median. The median thickness of the breasts was 4.1 cm (range, 2.0–7.2 cm). Breast thinner or thicker than the median were defined as thin breasts and thick breasts, respectively. Multiple comparison tests were performed for each group.

## RESULTS

This planning study included 51 patients with early stage breast cancer: 20 with right-sided breast cancer and 31 with left-sided breast cancer. The median age of the patients was 53 years (range, 26–76 years).

The Dmax, V100%, V95% and HI for each method are shown in Table 1. The average V100% with ASM was significantly higher than that with SSM and MSM (*P* = 0.0329 and 0.0003, respectively). The average Dmax, V95% and HI did not differ significantly between these three methods.

**Table 1.** Average of dose parameters of PTV for each method

	SSM (± SD)	MSM (± SD)	ASM (± SD)
Dmax	52.5 (± 0.7)	52.2 (± 0.6)	52.2 (± 0.7)
V100%	52.6 (± 16.7)	48.7 (± 14.9)	60.3 (± 14.2)
V95%	93.7 (± 4.2)	93.2 (± 4.1)	94.1 (± 3.5)
HI	0.112 (± 0.020)	0.108 (± 0.018)	0.110 (± 0.017)

PTV = planning target volume, SSM = single pair of subfields method, MSM = multiple pairs of subfields method, ASM = alternate subfields method, SD = standard deviation, Dmax = maximum dose, V100% and V95% = percentage of the PTV volume receiving 100% and 95% of the prescription dose, HI = homogeneity index.

The Dmax, V100%, V95% and HI for each method in the thin breast group are shown in Table 2. The average V100% with ASM was significantly higher than that with MSM (*P* = 0.0127). The average V100% with SSM was also significantly higher than that with MSM (*P* = 0.0360). The average Dmax, V95% and HI did not differ significantly between these three methods.

**Table 2.** Average of dose parameters of PTV for patients in the thin breast group for each method

	SSM (± SD)	MSM (± SD)	ASM (± SD)
Dmax	52.2 (± 0.6)	51.9 (± 0.5)	51.9 (± 0.6)
V100%	54.2 (± 16.6)	43.3 (± 15.2)	57.3 (± 15.8)
V95%	93.3 (± 3.4)	91.8 (± 4.3)	93.6 (± 3.2)
HI	0.115 (± 0.021)	0.112 (± 0.020)	0.112 (± 0.018)

PTV = planning target volume, SSM = single pair of subfields method, MSM = multiple pairs of subfields method, ASM = alternate subfields method, SD = standard deviation, Dmax = maximum dose, V100% and V95% = percentage of the PTV volume receiving 100% and 95% of the prescription dose, HI = homogeneity index.

The Dmax, V100%, V95% and HI for each method in the thick breast group are shown in Table 3. The average V100% with ASM was significantly higher than that with SSM and MSM (*P* = 0.0120 and 0.0208, respectively). The average Dmax, V95% and HI did not differ significantly between these three methods.

**Table 3.** Average of dose parameters of PTV for patients in the thick breast group for each method

	SSM (± SD)	MSM (± SD)	ASM (± SD)
Dmax	52.9 (± 0.6)	52.5 (± 0.6)	52.6 (± 0.6)
V100%	51.0 (± 17.0)	54.4 (± 14.5)	63.4 (± 11.7)
V95%	94.0 (± 4.9)	94.7 (± 3.3)	94.6 (± 3.9)
HI	0.109 (± 0.020)	0.104 (± 0.015)	0.107 (± 0.016)

PTV = planning target volume, SSM = single pair of subfields method, MSM = multiple pairs of subfields method, ASM = alternate subfields method, SD = standard deviation, Dmax = maximum dose, V100% and V95% = percentage of the PTV volume receiving 100% and 95% of the prescription dose, HI = homogeneity index.

## DISCUSSION

Breast tangential radiotherapy using the FIF technique has been widely reported. However, the methods for the FIF used in these studies vary. The simplest method is the addition of a single pair of subfields [4–8, 16, 17]. This method was classified as SSM. SSM is often used in studies conducted in Asian countries such as Japan. In addition, radiotherapy planning for SSM requires less time because only a few subfields need to be generated. However, the method most commonly reported is the one in which multiple pairs of subfields are used [9–15, 18–23]. This method was classified as MSM. The planning time is longer for this method because of the high number of subfields. In terms of the number of the subfields, another method introduced by the MD Anderson Cancer Center group at the University of Texas employs fewer subfields than MSM but more than subfields than SSM [24, 25]. The most significant feature of this method is the recalculation each time when creating subfields, and the addition of subfields alternately. This method was classified as ASM.

The Dmax did not differ significantly among the three methods, and the degree of reduction of the hot region was the same for all methods. The average V100% was significantly higher with ASM than with SSM or MSM. In the thin breast group, the average V100% was significantly higher not only with ASM but also with SSM than that with MSM. For patients with not very large breasts, it is difficult to considerably increase the Dmax and create hot regions. Creation of many subfields for such patients may result in the production of cold regions. In the thick breast group, the average V100% was significantly higher with ASM than with SSM or MSM.

The ASM outperformed the SSM and MSM for two possible reasons. The first is that the number of subfields used is more suitable for the population under study. When the number of subfields is large, the dose to the PTV decreases, but when the number of subfields is small, the full range of advantages of the FIF cannot be fully obtained. The second reason originates from the characteristics of the ASM. The biggest advantage of the ASM is its ability to perform dose calculation each time a subfield is added. The subfields were formed in order to shield the areas of the breast receiving any dose cloud on BEV. However, dose reduction also occurs at a site close to the area that is blocked. When the subfields were paired, the dose reduction region overlapped and was enhanced. In contrast, as a rule, in order to avoid the creation of a pair of subfields in ASM, the dose reduction region in the circumference of a block domain does not overlap. Moreover, by performing dose calculation just before the creation of a subfield, the subfield reflecting this influence can also be created, and the use of an excessive number of blocks can be avoided. For these reasons, the unintended dose reduction was small and the V100% had a good value. Thus, ASM resulted in better dose distribution regardless of the breast size. In addition, radiotherapy planning with ASM required a relatively short time, and thus, ASM was considered the most preferred method. Of note, patients in the thin breast group derived similar benefit with ASM and SSM. Because SSM is the simplest of the three methods, it should be the method of choice for patients with small breasts. We were unable to confirm the usefulness of MSM in this study. All patients included in this study were Japanese, and the average breast size was considered small compared with that of patients in Western countries. For patients with breasts larger than those included in this study, MSM may be useful.

## FUNDING

This work was supported by Gifu University.
